# Regular Meeting of Fulton County Medical Society, October 7, 1915

**Published:** 1915-09

**Authors:** Allen H. Bunce


### REGULAR MEETING OF THE FULTON COUNTY MEDICAL SOCIETY, OCTOBER 7, 1915.

DR. ALLEN H. BUNCE

Dr. G. D. Ayer exhibited a case from which he had removed a tumor of the orbit of the right eye. The first symptom noticed with reference to this case was that his eyes were found to be crossed by his mother. Upon examination, Dr. Ayer discovered the tumor as the cause of the crossed eyes and removed it. The boy's eyes are now normal. His vision is 20/30. A pathological examination of the tumor showed it to be an endothelioma. These tumors are very rare occurring only in one to six or ten thousand cases of this kind.

Dr. M. T. Davis read a paper on "Some Observations on the Use and Abuse of Opium."

In discussing the paper Dr. Duncan remarked that it was a very timely contribution, particularly at this time. Dr. Duncan has had one case that he has been able to cure of the opium habit. She has now been off the drug six or eight months.

Dr. Frank Wells inquired of Dr. Davis why he used such small doses of hyoscine. Dr. Wells is in the habit of using 1/00 of a grain.

In closing Dr. Davis said the reason he used small doses was because he has found that many patients have an idiocycrasy for the drug, especially alcoholics. He has found that 1/600 of a grain controls the craving for morphia in practically all cases. In discussing the difficulty in keeping the drug from patients he reported that he had one patient where the nurse discovered that the patient had morphia wrapped in wax paper in the vagina.

Dr. Roy Blosser exhibited: "Two Cases of Probable Sprue."

In discussing these cases Dr. Steve Harris said that Sprue does occur in Georgia to a certain extent. Dr. Harris, himself, had Sprue in 1919 in the Philippines. Dr. Harris has noted that in Sprue white yeasty stools are found. Cases of Sprue do recover.

Dr. Elmore Thrash said that several years ago he saw many cases which he diagnosed as Sprue. He thinks there is no sharp line of division between Sprue and Pellagra. He considers Sprue artypical Pellagra just as we have artypical cases of Typhoid. The history and symptoms of the present two cases are those of Pellagra.

Dr. Steve Harris remarked that there are no mental symptoms or skin eruptions in Sprue. Sprue does not recur.

In closing, Dr. Blosser said that Sprue is different from Pellagra in several respects. He wishes to follow these cases very closely.

Dr. Dunbar Roy read a paper: "Paresis of the Soft Palate following Tonsillar and Adenoid Operation—Remarks on the Tonsils and Adenoids."

Dr. R. R. Daly said that the paper of Dr. Roy came as a timely warning. Many cases are referred to the Throat Specialist who need no operation. He considers that the surgical tonsil may be studied from two standpoints: (1) the tonsil which presents some abnormality within itself, (2) the tonsil which obstructs the eustachian tube. Dr. Daly has noticed that patients from 14 to 20 years old, with singing voices sometime have difficulty in learning to phonate properly after a tonsilectomy. It takes some cases as long as a year to learn to use their voices properly again. However, all of his cases have learned to use their voices again successfully. He reported a case in which he did a plastic operation on the posterior pillar of the tonsil. He found that he is able to do his work very accurately by guidng his instruments by the sense of touch. Dr. Daly advises a small, sharp curet for the removal of adenoids. The ordinary adenotome is too large and its use is liable to injure the tissues.

Dr. S. T. Barnett said he regarded the paper of Dr. Roy as a very timely warning. He thinks that unnecessary tonsil and adenoid surgery may result in permanent injury to the patient.

Dr. Floyd McRae reported a case which had been called to his attention where the surgeon was sued for $20,000 damages on account of paralysis of the soft palate following a tonsil operation.

Dr. W. N. Adkins said that paralysis of the soft palate sometimes follows a mild case of diphtheria and he wonders if infection doesn't play an important part in the causation of the paralysis.

In closing Dr. Roy said that he considered the removal of tonsils and adenoids a major operation which should always be done in a hospital under a general anesthetic. Accidents are liable to happen even after the work of the most careful men, however, by taking into consideration the seriousness of the operation and by using the proper care bad results are less likely to follow.

Dr. S. T. Barnett reported a case of facial paralysis in the new born following a forceps delivery. In looking up the literature on the subject Dr. Barnett has found that this may occur when forceps are not used. This is the first case he has had in his practice and reports that the case is improving considerably.

Dr. Floyd McRae reported two cases of stone in the common duct where the cachexia simulated that of malignant diseases. The first had the beginning of the trouble seven years ago, and has had numerous attacks since that time. The attacks come on as follows: pain-chill-fever-sweat. The second case had been jaundiced 18 months and the liver was extended to near the crest of the ileum.

Dr. Barnett said that Pellagrins with gall bladder symptoms are bad surgical risks.

Dr. Thrash said that these cases should be examined very carefully before any operative procedure is undertaken. Tuberculosis is very often overlooked in surgical cases only to have it develop very acutely after the operation.

Dr. McRae has operated on a number of cases of pellagra with good results.

